# Regulation of Mitochondrial Structure and Dynamics by the Cytoskeleton and Mechanical Factors

**DOI:** 10.3390/ijms18081812

**Published:** 2017-08-21

**Authors:** Erzsébet Bartolák-Suki, Jasmin Imsirovic, Yuichiro Nishibori, Ramaswamy Krishnan, Béla Suki

**Affiliations:** 1 Department of Biomedical Engineering, Boston University, Boston, MA 02215, USA; jasmin.imsirovic@gmail.com (J.I.); pyxf4fnk@s.okayama-u.ac.jp (Y.N.); bsuki@bu.edu (B.S.); 2 Department of Cardiovascular Physiology, Graduate School of Medicine, Dentistry and Pharmaceutical Sciences, Okayama University, Okayama 700-8558, Japan; 3 Center for Vascular Biology Research, Beth Israel Deaconess Medical Center, Harvard Medical School, Boston, MA 02215, USA; rkrishn2@bidmc.harvard.edu

**Keywords:** fission, fusion, bioenergetics, network, stiffness

## Abstract

Mitochondria supply cells with energy in the form of ATP, guide apoptosis, and contribute to calcium buffering and reactive oxygen species production. To support these diverse functions, mitochondria form an extensive network with smaller clusters that are able to move along microtubules aided by motor proteins. Mitochondria are also associated with the actin network, which is involved in cellular responses to various mechanical factors. In this review, we discuss mitochondrial structure and function in relation to the cytoskeleton and various mechanical factors influencing cell functions. We first summarize the morphological features of mitochondria with an emphasis on fission and fusion as well as how network properties govern function. We then review the relationship between the mitochondria and the cytoskeletal structures, including mechanical interactions. We also discuss how stretch and its dynamic pattern affect mitochondrial structure and function. Finally, we present preliminary data on how extracellular matrix stiffness influences mitochondrial morphology and ATP generation. We conclude by discussing the more general role that mitochondria may play in mechanobiology and how the mechanosensitivity of mitochondria may contribute to the development of several diseases and aging.

## 1. Introduction

Eukaryotic cells maintain a complex internal structure to perform specialized tasks such as migration, contraction, and cell division, as well as to respond to various chemical and mechanical cues from the environment. All these activities require energy that is primarily produced by the mitochondria in the form of ATP via the process of oxidative phosphorylation. Beyond this central role, mitochondria also guide apoptosis and contribute to calcium buffering and reactive oxygen species (ROS) production [1].

To support its diverse functions, mitochondria form an extensive network inside the cell with smaller mitochondrial clusters that have the ability to move along the cytoskeleton aided by motor proteins [2]. Furthermore, the network itself is highly dynamic in that it is constantly undergoing fission and fusion [3,4]. These processes are essential for both the integrity of the cell and the survivability of the organism. Indeed, genetically knocking out proteins responsible for fission [5] as well as fusion [6] in mice produces embryos which die before birth. Although there is extensive literature on mitochondrial network properties and dynamics [4,7,8,9,10,11], less is known about how other intracellular structures such as the cytoskeleton [12] and extracellular mechanical factors such as the stretching of the cells [13] affect mitochondrial functions. Since the effects of stretch on the cell are transmitted primarily via the cytoskeletal networks to which mitochondria are attached, it is conceivable that both the cytoskeletal organization and mechanical factors in general influence mitochondrial network structure and function.

The goal of this review is to present an overview of two important regulators of the mitochondria, the intracellular cytoskeleton and the extracellular mechanical factors. One important mechanical factor is the deformation that the cell is exposed to in the body during normal tissue function. It has recently been found that the in vivo natural dynamic nature of stretch pattern helps maintain general mitochondrial function [14]. Additionally, since the mechanical stiffness (defined loosely as the response in stress to a unit change in strain of the material) of the extracellular matrix (ECM) has emerged as a major regulator of many cell functions [15], it is important to address the question of whether ECM mechanics also influences mitochondrial function.

The organization of this review is as follows. We first briefly review the morphological features of the mitochondria with an emphasis on how specific network properties govern function. We then review the relationship between mitochondria and the cytoskeletal structures, focusing on the interactions between the two networks. Next, we discuss how stretch and its dynamic pattern affect mitochondrial structure and function. Finally, we present some preliminary data on how ECM stiffness influences mitochondrial morphology and ATP generation. We conclude by discussing the more general role that mitochondria may play in mechanobiology and how the mechanosensitivity of mitochondria may contribute to the development of several diseases and aging.

## 2. General Mitochondrial Structure and Function

### 2.1. Intra-Mitochondrial Structure and ATP Generation

Mitochondria are thought to have originated from free-living alpha-proteobacteria that developed a symbiotic relationship with the host cell [16]. There is now overwhelming phylogenic evidence for this scenario [17], supported by the facts that mitochondria have their own DNA, denoted by mtDNA, and that several mitochondrial proteins also have bacterial origin [18]. Similar to bacteria, these organelles are bound by an outer membrane and an inner membrane. The outer membrane allows the exchange of metabolites between the inner membrane and cytosol, but can also seal the mitochondria from releasing harmful agents into the cytosol such as ROS and mtDNA [19,20]. Nevertheless, in subtoxic amounts, mitochondrial ROS serve as signaling molecules following release into the cytosol [21].

**Figure 1:**
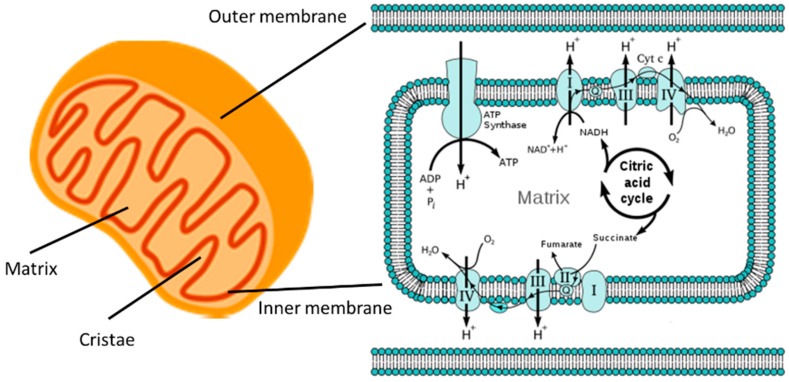
The mitochondria are the powerhouses of the cell. Left: A cartoon of a mitochondrion showing its outer and inner membranes, the cristae, and the matrix. Fat and sugar enter the mitochondria through channels of the outer membrane. Right: The Krebs, or citric acid, cycle feeds the chain of respiratory complexes I through IV which create an electrical and proton (H^+^) gradient, the electromotive force across the inner membrane. ATP synthase utilizes the electromotive force to generate ATP from ADP and inorganic phosphate (P_i_) (Right image from Wikipedia [24]).

The inner membrane consists of distinct morphological regions including the membrane boundary, the cristae junctions, and the cristae [22]. The cristae are the invagination of the inner membrane that significantly increase the surface area; this is also where proteins of the electron transport chain (ETC) are located. The cristae morphology is organized by the mitofilins that accumulate between the inner and outer membranes [23]. Inside the inner membrane is the mitochondrial matrix where the Krebs cycle takes place. The Krebs cycle feeds NADH and FAD into two transmembrane proteins, the respiratory complex I and II, respectively. Energetic electrons move along the respiratory complexes of the ETC while protons are transported from the matrix into the intermembrane space, leading to a build-up of charge and proton gradient across the inner membrane, called the electromotive force, acting like a battery which stores electrochemical energy. The terminal component of the ETC is the ATP synthase, which uses the electromotive force to attach an inorganic phosphate group to ADP and produces ATP. The entire process is known as oxidative phosphorylation. A fraction of the ATP is subsequently utilized by the mitochondria and the rest is released into the cytoplasm as a form of chemical energy for various cellular processes (Figure 1).

### 2.2. Mitochondrial Dynamics

The above picture paints mitochondria as isolated organelles utilizing food from their immediate neighborhood within the cell. However, it is now known that mitochondria are highly dynamic and interacting organelles [20]. Inside the mitochondria, the cristae can significantly remodel themselves in response to environmental cues and stresses. For example, the availability of energy-rich substrates modulates mitochondrial cristae architecture [25], which in turn drives respiratory complex assembly and mitochondria-dependent cell growth [26]. The dimer form of ATP synthase dissociates during aging, which is followed by a loss of cristae invagination [27] perhaps suggesting a correlation between ATP synthase and cristae structure. However, the primary regulatory protein responsible for cristae maintenance is the dynamin-related GTPase protein Optic Atrophy 1 (OPA1), which appears to have distinct functions in mitochondrial fusion and in cristae remodeling, at least during apoptosis [28].

**Figure 2:**
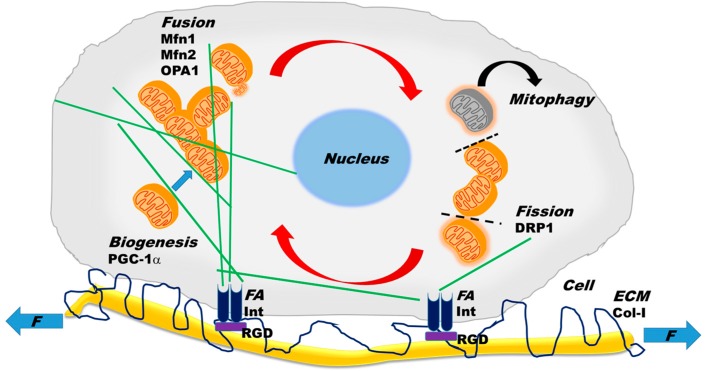
Intracellular and extracellular processes contributing to mitochondrial structure and function. Intracellular mitochondrial (orange) dynamics include the processes of fusion, fission, mitophagy, and biogenesis. See text for explanation for how the various molecules such as OPA1, Mfn1, Mfn2, and DRP1 govern these processes. The grey mitochondrion is damaged and degraded by mitophagy. Green lines represent microtubules, along which small mitochondrial clusters can travel with the aid of motor proteins. The dashed line represents the site of fission and the red arrows indicate the cyclic nature of fission and fusion. Note that biogenesis increases mitochondrial volume and is regulated by the peroxisome proliferator-activated receptor γ coactivator (PGC-1α). Cells are connected to the ECM (extracellular matrix) and exposed to external mechanical forces (F) at focal adhesions (FA), involving integrin receptors (Int) on the cell surface and Arg-Gly-Asp (RGD) binding sites on collagens fibers (Col-I). The cytoskeleton is linked to FAs and therefore mechanical forces from the ECM are transmitted to the mitochondria.

Individual mitochondrial clusters are also capable of fusion (Figure 2), forming large organelles that range in size from a few tens to thousands, depending on the cell type. These clusters form a dynamically interconnected reticular network which can spread over the entire cell volume. The network elements have a cylindrical shape with a diameter of a few hundred nanometers. The formation of this network by fusion involves merging both the outer and inner membranes of two separate mitochondrial clusters, and their content including mtDNA becomes completely mixed within 12 h [29]. The outer membrane fusion is governed by the mitofusins Mfn1 and Mfn2 [6], whereas the inner membrane fusion is regulated by OPA1 [30]. Interestingly, while the fusion of outer membranes is independent of oxidative phosphorylation, the inner membrane fusion requires enzymatic cleavage of OPA1 which is stimulated by high membrane potential [31], suggesting that only healthy and active mitochondria can fuse properly.

The large mitochondrial clusters can also undergo fission (Figure 2), whereby a mitochondrial cluster splits into two or more clusters. Following fission, the smaller clusters may become nearly spherical with diameters of about a few hundred nanometers. Fission is necessary for the distribution of mitochondria during cell division and embryonic growth [32]. However, if fission is not controlled and balanced by fusion, the network becomes too fragmented which leads to glucose oxidation, mitochondrial inner membrane potential decline, and hence the downregulation of ATP production [8]. The process of fission is coordinated by a set of events to which components of the cytoplasm, cytoskeletal elements, as well as organelles can contribute in three steps: (1) marking the fission site; (2) assembly of cytosolic dynamin-related protein 1 (DRP1) into a superstructure at the fission site; and (3) constriction of the membranes at the fission to split the mitochondrial cluster into daughter clusters [20,33].

Fission and fusion rates are believed to be in well-coordinated balance [34]. Specifically, the interaction of two heptad-repeat regions of Mfn2 inhibits fusion, whereas their binding to DRP1 promotes fusion [35]. Furthermore, the long form of OPA1 is able to mediate fusion, while the enzymatically processed short form of OPA1 promotes fission [36]. Interestingly, the latter study also found that the short form of OPA1 co-localizes with contact sites of the mitochondria with the endoplasmic reticulum (ER). Mitochondrial fission is also mediated by physical interactions with narrow ER tubules. When aquaporin water channels allow swelling of the ER tubules, the constriction of the tubules contributes to the mechanical force required for fission [37]. Indeed, fission occurs at the contact sites between mitochondria and ER tubules and the constriction is initiated before the recruitment of DRP1 [38]. Further roles of the ER and mitochondrial tethering are discussed in more detail elsewhere [34]. Two additional processes contribute to the full dynamics of mitochondria; biogenesis, the production of mitochondrial content both by the nucleus and the mitochondria, and mitophagy, the degradation and elimination of damaged mitochondria (Figure 2).

### 2.3. Mitochondrial Network Properties

The organization and apparent complexity of the mitochondrial clusters prompted more quantitative investigations of the structure from a network perspective using fluorescence imaging and mathematical analyses. One study reported that fission and fusion are not fully independent and that they may form a cycle (Figure 2) in which the probability that fission is followed by fusion and fusion is followed by fission is ~0.8 [39]. Furthermore, in neuronal cells, fission was found to be driven by mitochondrial cluster length, whereas fusion was governed by cluster motility in such a way that the total rates of fission and fusion were matched and maintained in a homeostatic state [39]. It is also important to note that the precise rates and probabilities depended on the cell type. The energetic state of mitochondria is critically linked to its structure, since dissipating the inner membrane potential, inhibiting complex III and complex V (ATP synthase) of the ETC, or reducing cytosolic ATP by inhibiting glycolysis all reduced fission and fusion rates, attenuated motility as measured by the diffusion coefficient, decreased the probability of bursting cluster motion, and increased mitochondrial fragment numbers [40].

To characterize the structure of individual clusters, one can measure the length of the cluster along the backbone [39], compute an apparent aspect ratio (AR = ratio of major to minor axis of an ellipse fit to the cluster shape) or use the form factor (FF = perimeter squared divided by the product of 4π and the area) [41]. The AR characterizes how globular a cluster is, whereas FF is related to the extent of branching along the backbone of the cluster. The entire mitochondrial network complexity can be quantified by the fractal dimension D_f_ that is related to the space filling capacity of the network [7,14]; if in two dimensions (2D) D_f_ is close to 1, the network consists mostly of lines, and if D_f_ is close to 2, the network tends to be a compact 2D object such as a filled circle. To study what governs the structural properties of the mitochondrial network, it is useful to image some fluorescent labels associated with the mitochondria and compute the above indices before and after applying various stimuli or inhibitors. For example, mitochondrial average cluster size in vascular smooth muscle cells (VSMC) in culture without stretch was found to depend on a number of inhibitors including blebbistatin, an inhibitor of non-muscle myosin II, dynasore, an inhibitor of DRP1, and paprotrain, an inhibitor of mitotic kinesin-like protein 2 that allows mitochondria to move along microtubules [14]. Furthermore, chronic inhibition of complex I with rotenone in human skin fibroblasts as a model of mitochondrial dysfunction significantly increased FF, suggesting that hyperproduction of ROS leads to mitochondrial outgrowth [42].

When the entire mitochondrial network is considered, a global picture arises that this network spans the cell efficiently, which is most likely an evolutionary consequence of optimal spatial distribution of energy supply in the form of ATP. This global network can be characterized by its connectivity using percolation theory [43]. Consider a simple lattice in which each neighboring site is occupied with probability *p*. A cluster is defined as a set of connected occupied sites. As *p* increases from 0, defining a completely empty and disconnected lattice, to 1, a fully connected lattice, the size of clusters gradually increases. There is a point at which a large cluster spans the lattice, providing full connectivity from one side to the other. The transition from a disconnected lattice to one that includes a connected cluster spanning the system occurs when *p* crosses a critical percolation threshold, denoted by *p*_c_. The structure of the percolation cluster at *p* = *p*_c_ is a self-similar fractal. Thus, such a percolation transition should occur when a critical density of mitochondria is linked into a global network via fusion. To test this idea, Aon et al. [7] showed in isolated ventricular myocytes that the mitochondria form a network near the critical point, and the fractal properties of the network agree well with a percolation-like mitochondrial network. Even more interesting is to consider how signaling through such a network occurs. When mitochondria accumulate sufficient amounts of ROS that exceed a threshold, a small additional release of ROS, produced locally by leakage of the ETC, triggers a ROS wave that spreads through the connected cluster, first depolarizing the inner membrane potential, followed by a transition to oscillations. The biological consequences, argued the authors [7], are that a transition to the oscillatory behavior may destabilize cardiac “action potential repolarization in the whole heart, suggesting that criticality at the microscopic level may be translated into the death of the organism”. This scenario suggests that mitochondria can signal across cells, reaching the tissue level to influence the fate of organs and the organism.

## 3. Cytoskeletal–Mitochondrial Interactions

In the previous sections, we presented a brief overview of the structure, dynamics, and regulation of mitochondria. In this section, we discuss the interactions between mitochondria and the components of the cytoskeleton (Figure 2). The functional roles of the cytoskeleton, a filamentous protein network, are to provide cells with resistance to deformation, allow shape change during movement, transport cargo including mitochondria, and mechanotransduction, the conversion of mechanical stimuli to signaling [44]. Additionally, both internal and external physical forces can be transmitted through the cytoskeleton to other organelles including the nucleus and the mitochondria. Interestingly, the cytoskeletal network itself can respond to external forces by exhibiting hysteresis and memory, and long-lived cytoskeletal structures can be epigenetically inherited by future generations following cell division [44]. Because of this fundamental mechanical role of the cytoskeleton in general cell behavior, our focus will be mostly on the mechanical aspects of the relationship between the cytoskeletal and the mitochondrial networks.

The cytoskeleton is composed of three main types of polymers: actin filaments, microtubules, and intermediate filaments. These filaments form interconnected networks with the help of cross-linkers, motor proteins, and stabilizers. The amount and structural organization of these networks determine the shape and mechanics of the cell. These networks can also respond to external forces by reorganizing their network structure and communicating mechanical forces to each other and to various organelles. The reorganization often involves polymerization and depolymerization, regulated by factors such as nucleation-promoting factors, which initiate growth, capping proteins, which stop polymerization, polymerases, which enhance polymer growth and depolymerizing factors, which disassemble the filaments and networks [44]. All three cytoskeletal networks have been associated with various mitochondrial functions.

### 3.1. Interactions of Mitochondria with the Actin Cytoskeleton

An early study suggested a spatial co-localization of gamma actin with skeletal muscle cell mitochondria [45], whereas a later study showed that in chick sympathetic neurons, mitochondria can move along the axon in both directions and their motility requires either microtubules or actin, depending on which cytoskeletal network is present [46]. However, it was subsequently reported that in axons and dendrites, mitochondria showed a preferred movement along microtubules, although some limited movement along actin was possible [47]. While mitochondrial movement requires actin in plants and fungi or microtubules in mammalian cells, actin also helps the immobilization of mitochondria in neurons at locations where ATP is needed [48], by strengthening its Ca^2+^-dependent interaction with actin [49]. Actin also participates in the redistribution of the mitochondrial network during mitosis. The transport of mitochondria towards the daughter cell at the end of mitosis is promoted by the cell cycle-dependent recruitment of Cenp-F, a cytoskeleton-associated protein, by a mitochondrial protein called Miro [50].

The cortical actin structure depends on the availability of both ATP and non-muscle myosin II, and this myosin cross-linked actin network determines the stiffness of the cell [51]. Inhibiting non-muscle myosin II indeed alters the cortical actin [52], but this inhibition also reduces the average mitochondrial cluster size in VSMCs [14]. However, mitochondria also influence many actin-related cell functions. For example, since ATP is mostly produced by the mitochondria, cell stiffness also depends on mitochondrial ATP production. The same applies for cell contraction. Indeed, it was shown recently in VSMCs and aorta rings that inhibiting the ATP synthase with oligomycin reduced active force generation [14]. Thus, there is a subtle and bi-directional relationship between cortical actin and mitochondrial structure that contributes to overall cell mechanical functions such as stiffness and contractility.

### 3.2. Microtubules Regulate Mitochondrial Function

Mitochondria have long been known to strongly interact with microtubules in many cell types [53]. Tubulins, both alpha and beta, were found to be localized in mitochondria and were closely associated with mitochondrial voltage-dependent anion channels (VDACs) [54]. The strategic localization of beta-tubulin II near VDACs allows it to regulate the so-called mitochondrial permeability transition [55], in which the pores, mostly VDACs, on the outer membrane open, leading to necrosis and apoptosis [56].

Microtubule filaments serve as railroad tracks upon which mitochondrial clusters travel within the cell, utilizing motor proteins (Figure 2) such as dyneins and kinesins, which allow movement toward microtubule’s minus and plus ends, respectively [57]. Disassembling microtubules completely eliminates mitochondrial motility [47], which is necessary for fusion and fission and hence for the maintenance of healthy mitochondrial structure and bioenergetics. Indeed, graph theoretical analysis and mathematical modeling provide evidence that besides the fusion and fission rates, mitochondrial structure is determined by the retrograde and anterograde movements and the balance between these rates is responsible for the heterogeneous distribution of mitochondria in the cell [58]. Microtubules also contribute to cell shape and stability by their ability to carry compressive forces [59]. External forces can alter cell shape, resulting in a reorganization of microtubules; indeed, cyclic uniaxial stretch changed cell orientation and microtubule structure [60], which can affect mitochondrial cluster and network properties and hence ATP production. This phenomenon is further discussed below in relation to how external mechanical forces influence mitochondria.

### 3.3. Contribution of Intermediate Filaments

There is evidence that intermediate filaments also contribute to mitochondrial structure and function [12]. For example, plectin, a cytoskeletal crosslinker, is spatially associated with desmin, an intermediate filament, which is co-localized in an ordered fashion with mitochondria along the length of the sarcomere of striated muscle, suggesting the possibility that these proteins contribute to the branching pattern of mitochondria [61]. Vimentin is another intermediate filament which is also associated with mitochondria, since vimentin-null cells showed mitochondrial fragmentation and disorganization perhaps by modulating the association of mitochondria with microtubules [62]. The fact that vimentin plays a role in cell mechanics as well as protects the cell against compressive stresses [63] further suggests the possibility that external mechanical stresses may regulate mitochondrial structure and function.

## 4. Mechanobiology of Mitochondrial Structure and Function

Nearly all cell types in the body are exposed to mechanical factors such as shear stress, external pressure, tensile stress, and the stiffness of the surrounding ECM. These mechanical factors affect cell function via the interaction of cell surface receptors such as integrins at focal adhesions with binding sites on ECM fibers, such as Arg-Gly-Asp (RGD) on collagen type I (Figure 2). Cells have co-evolved with the ECM to respond to such stimuli and continuously attempt to maintain a homeostatic state with the ECM. Indeed, vascular endothelial cells are sensitive to shear stress [64], whereas kidney cilia [65], bone [66], cartilage [67], and eye [68] cells respond to pressure. Muscle contraction generates stresses on muscle cells [69] as well as nerve cells [70]; cyclic variations in blood pressure in the arteries produce circumferential stresses acting on VSMCs [71]; and breathing cyclically stretches all cells of the lung [72]. With regard to ECM stiffness, the best example is that stem cells are neurogenic on soft ECMs that mimic the brain, myogenic on stiffer ECMs that mimic muscle, and osteogenic on very stiff ECMs that mimic bone [73].

### 4.1. Effects of Transient and Monotonous Stretch

**Figure 3:**
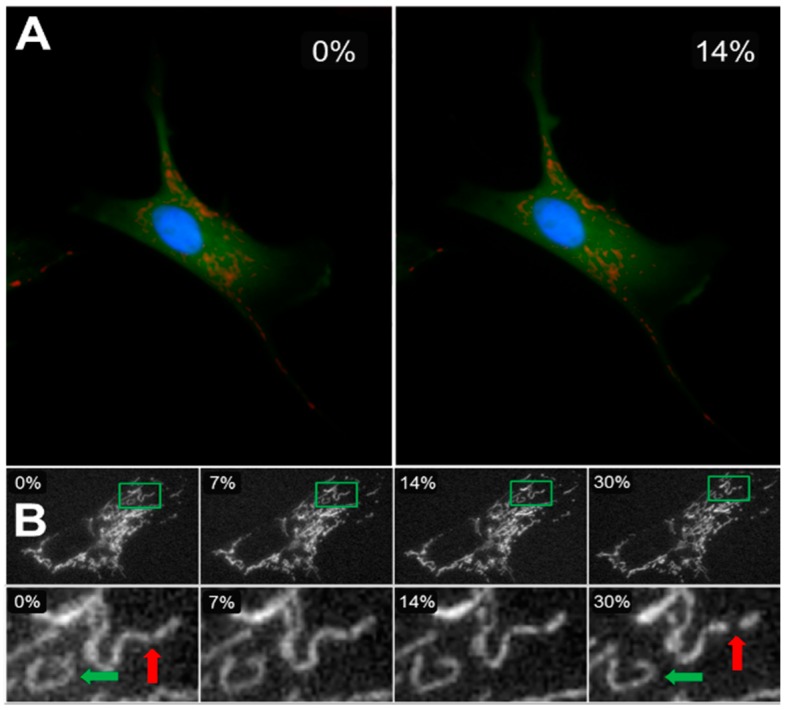
Cells were cultured on elastic membranes that could be stretched equibiaxially in a stretching device. (**A**) A cell labeled for cytosol (green), mitochondria (red: tetramethylrhodamine methyl ester, TMRM), and nucleus (blue) at 0% (left) and 14% (right) strains. (**B**) The top row shows the mitochondrial network of an entire cell imaged during constant strain application at increments of 0, 7, 14, and 30% change in membrane surface area. The bottom row shows the zoomed-in details of an individual cluster (green rectangle in top row) changing shape as higher strains are applied (green arrow), as well as a cluster undergoing fission and splitting into two smaller clusters (red arrow) [77].

Since the cytoskeleton is the primary load-bearing element that responds to all external mechanical stimuli, the strong link between the cytoskeletal and mitochondrial networks suggests that mitochondria should also be mechanosensitive. Nevertheless, mitochondrial responses to mechanical stimuli received attention only relatively recently. For example, in an in vitro sustained stretch model (24 h with 20% area strain) of abnormal mechanical milieu, cardiomyocytes underwent apoptosis because cytochrome c was released from mitochondria [74]. Mitochondrial membrane potential also declined, which seems to have resulted in mitochondrial fragmentation as seen in Figure 2B in Reference [74]. The authors concluded that the Bcl-2 proteins contributed to stretch-induced mitochondrial apoptosis. Respiratory muscle weakness was studied in intensive care units due to impairments in diaphragm contractility using a five-day mechanical ventilation model in piglets [75]. While mitochondrial content did not change, the activity of complex IV of the ETC decreased by 21%. Mechanical ventilation, however, takes away the natural variability in tidal breathing (see more details below) and hence this study suggests that long-term abnormal monotonous mechanical stimuli can result in specific molecular changes in the mitochondria. Cyclic mechanical strain increased ROS production in endothelial cells in an actin cytoskeleton-dependent manner [13]. Similarly, cyclic stretch upregulated ROS production in lung epithelial cell types in culture in a duration and amplitude-dependent fashion, suggesting that overdistension of the lung during mechanical ventilation may lead to mitochondrial ROS-induced lung injury [76]. Interestingly, the study also found direct evidence based on imaging that a global equibiaxial strain (17% strain applied to the elastic membrane on which cells were cultured) resulted in local stretching of the mitochondria with up to 32% linear strain. Furthermore, when lung fibroblasts were exposed to large transient equibiaxial stretches of up to 30%, mitochondria were seen to rupture (Figure 3) at discrete locations immediately after the stretch [77]. To visualize mitochondria, cells were labeled with tetramethylrhodamine methyl ester (TMRM), a dye whose intensity is related to the inner mitochondrial membrane potential [78] and hence to ATP production [79]. The results suggest that external mechanical stresses are capable of directly and immediately initiating fission. The reason for this is that the cytoskeleton is in a so-called pre-stressed state with tensile forces on actin fibers due to myosin motor activity [80], which primes the cytoskeleton for the rapid transmission of mechanical forces to long distances. Indeed, this is reminiscent of the fast and long-range mechanical force transmission from focal adhesions to the nucleus [81]. While the above studies confirm that external mechanical forces, both transient and long-term monotonous, can influence the structure and function of mitochondria, the internal mechanical microenvironment has also been shown to drive fission via an elastocapillary instability [82].

### 4.2. Fluctuations Influence Mechanotransduction

Mechanical stresses acting on tissues and cells in the body display significant variations and fluctuations. Beat by beat blood pressure changes exhibit significant variability, which is elevated in hypertension [83,84]. Breathing generates large breath-to-breath variability of tidal volume [85]. Since both the magnitudes and timing of mechanical stresses regulate the actual cellular signaling response [86], it is expected that fluctuations in the mechanical stimuli influence the details of mechanotransduction, a process called fluctuation-driven mechanotransduction (FDM) [87]. It is conceivable that over evolutionary time scales, FDM has become built into all mechanosensitive cellular processes. However, in standard laboratory conditions, mechanotransduction is studied using static or cyclic but monotonous stretch (MS). Several recent studies presented evidence that fluctuations in cycle-by-cycle strain or shear stress, called variable stretch (VS) or variable shear stress (VSS), respectively, fundamentally alter cellular behavior, including cytoskeletal organization, bioenergetics, and signaling [14,88,89].

**Figure 4:**
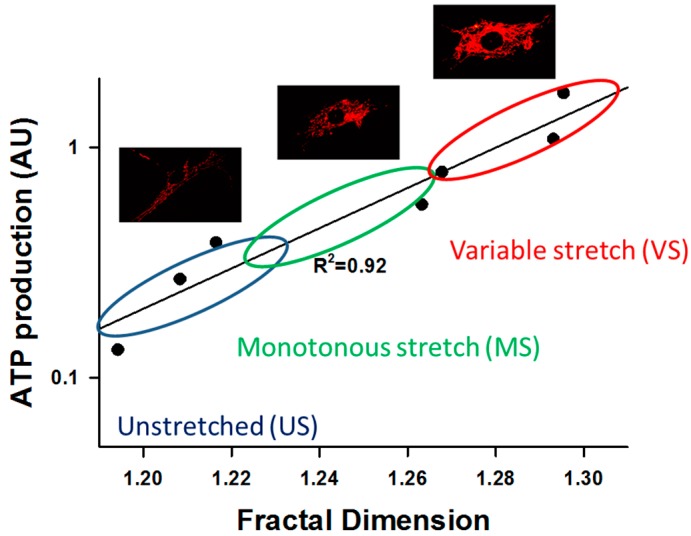
Relationship between complexity, measured by the fractal dimension D_f_, and function, assessed by a fluorescent dye (TMRM, see text) intensity that is related to ATP production rate in VSMCs. There is a linear relation between the log of ATP production and D_f_. The dots represent binned data from about 2000 cells showing unstretched control cells (US), 4 h of monotonously stretched (MS) cells (10% area strain at 1 Hz), and 4 h of stretching cells with a variable stretch (VS) pattern in which every cycle is different with the amplitudes uniformly distributed between 7.5% and 12.5% area strain. The US cells are in the lower left corner. These cells produce little energy and their mitochondrial fractal organization is the least complex. MS cells produce somewhat more energy and their D_f_ is also higher, whereas VS cells produce the most ATP and have the highest complexity in terms of their fractal organization. The images show mitochondrial networks corresponding to US, MS, and VS cells. ATP production rate is related to the intensity of red color. The results were obtained by reanalyzing the data from [14].

Recently, we reported that FDM directly affects mitochondrial structure and function. Specifically, ATP production rate assayed by visualizing TMRM in VSMCs cultured on elastic membranes and stretched equi-biaxially was twice as high following four hours of VS compared to MS [14]. Furthermore, VS also directly affected components and phosphorylation of the ETC complexes: ATP synthase as well as cytochrome c oxidase and its phosphorylated form were upregulated together with Mfn1 and Mfn2, but not DRP1. Interestingly, VS also induced mitochondrial biogenesis since the master regulator of biogenesis under external stimuli, the peroxisome proliferator-activated receptor γ coactivator [90], PGC-1α (Figure 2), increased compared to MS [14]. These biochemical shifts were accompanied by various structural changes such as increased organization of the actin, microtubule, and mitochondrial networks, characterized by their fractal dimension and coefficient of variation [14]. To test the possible mechanisms of FDM, inhibitors of actin polymerization, microtubule depolymerization, ATP synthase, focal adhesion kinase (FAK), or calcium availability were used. The results showed that both ATP production and mitochondrial cluster size were decreased, but VS maintained a higher membrane potential than MS. However, inhibitors of microtubule and vimentin assembly eliminated the membrane potential differences between MS and VS cells. Furthermore, inhibiting non-muscle myosin II, DRP1, which regulates fission [91], or the mitotic kinesin-like protein 2 reduced membrane potential in VS cells to the levels in MS cells. Finally, the functional consequence of FDM-induced ATP production was an increase in myosin light chain phosphorylation both in VSMCs in culture and aorta rings, which in turn resulted in a higher contractile force generation during VS in aorta rings [14]. Thus, VSMCs are capable of utilizing fluctuations in their mechanical environment and the extracted energy surplus manifests in increased chemical energy stored in ATP as a result of the reorganization and interaction of the cytoskeletal and mitochondrial networks. This is a structure-complexity-function relationship that arises from macroscopic mechanical fluctuations producing changes in cytoskeletal and mitochondrial network complexity, which regulates oxidative phosphorylation and enhances bioenergetics. Figure 4 compares the relation between network complexity and ATP production for unstretched (US), MS, and VS cells. To put this in perspective, US cells produce and consume the least ATP, while VS cells produce and consume the largest amount of ATP. Thus, US cells are the closest to while VS cells are the furthest away from thermodynamic equilibrium, because they are able to harness energy from environmental fluctuations to charge mitochondria, the battery of life. This far-from-equilibrium operation is supported by a higher complexity of the mitochondrial network structure. Nevertheless, it remains to be seen if FDM has similar effects in other cell types.

### 4.3. Effects of ECM Stiffness on Mitochondria

There is little data in the literature related to whether ECM stiffness affects mitochondrial structure and function. Recently, it was reported that in cardiac myocytes, baseline metabolism is influenced by ECM stiffness and, even more interestingly, the ability of cells to adapt to metabolic stress is regulated by both ECM stiffness and fiber alignment [92]. To complement these findings, below we present preliminary data that suggest a weak dependence of mitochondrial structure and function on ECM stiffness in VSMCs in culture, in qualitative agreement with the result in cardiac myocytes.

**Figure 5:**
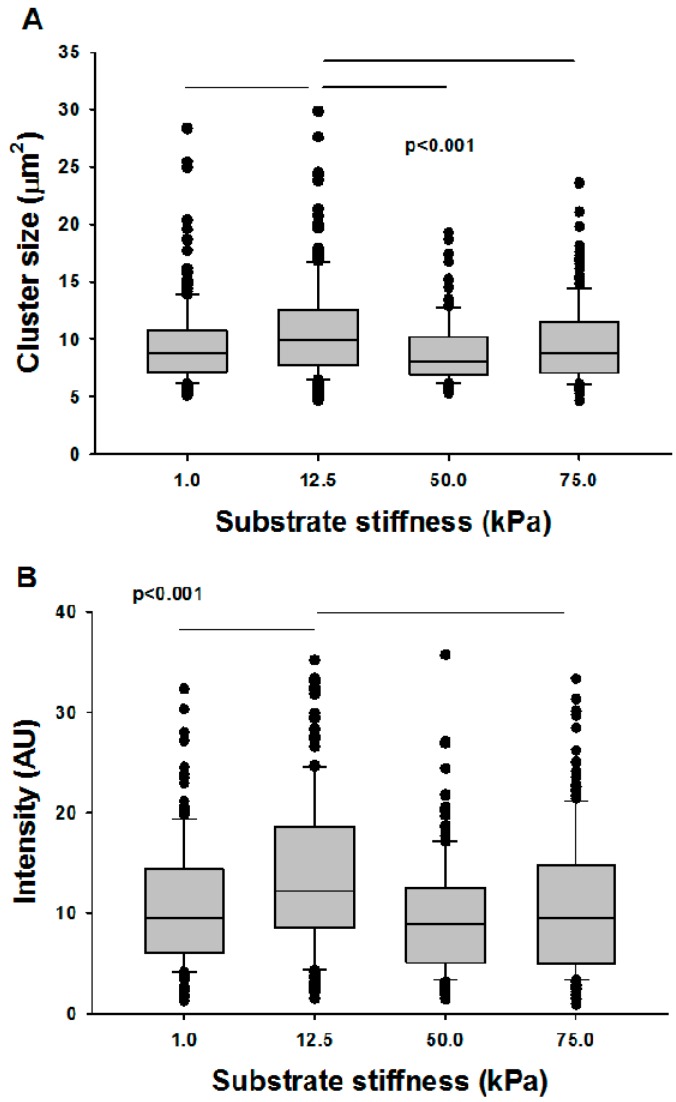
Mitochondrial structure-function relations as a function of substrate stiffness. VSMCs were seeded on substrates of different stiffness, labeled with TMRM, and cluster sizes and mean intensities were measured. The horizontal lines in the boxes are the median, the box represents the 25th percentile, and the horizontal bars are the 75th percentile of the data. The symbols are data outside the 75th percentile. (**A**) Cluster sizes were stiffness dependent, but only the clusters on 12.5 kPa stiffness were different from the rest. (**B**) Intensities were also stiffness dependent, and again only data on 12.5 kPa stiffness were different from the rest.

To test the effects of substrate stiffness on mitochondrial structure and function, the stiffness of an elastic gel (NuSil^®^ 8100, NuSil Silicone Technologies, Carpinteria, CA, USA) was characterized for different ratios of the polymer and its crosslinker [93]. Ratios of 1:1, 1:2, and 1:5 polymer to crosslinker were mixed and placed into an oven at 70 °C for 24 h. Stiffer gels were obtained by adding Sylgard 184 (Dow Corning, Auburn MI, USA) mixed at 1:10 ratio to the 1:1 NuSil at 20% and 33% by weight. Gel stiffness was determined using a uniaxial stretching device (*n* = 5 for each stiffness) as described previously [94]. The dimensions of the gel were measured before attachment into a stretching device, which imposed a known displacement and measured the resulting force. Stress and strain were calculated using the measured force and dimensions of the sample and the slope of a straight-line fit was taken as the stiffness. New gel layers were then prepared and ligated with type I collagen (Advanced Biomatrix, Carlsbad, CA, USA). VSMC isolated from bovine thoracic aortae were seeded on gels with nominal stiffness values of 1, 12.5, 50, and 75 kPa, and cells were subsequently labeled with TMRM. As Figure 5 demonstrates, the effects of stiffness on mitochondrial cluster size was statistically significant (*p* < 0.001), though moderate, showing at most 18% difference in median values between cells seeded on 12.5 kPa and the rest. The effect of stiffness on TMRM intensity was also significant (*p* < 0.001) and more pronounced, with the maximum intensity also occurring at 12.5 kPa and the largest difference among groups being 27%. It is interesting to note that 12.5 kPa is close to the in vivo stiffness of the vascular wall [95], and it is tempting to conclude that the organization and function of mitochondria in VSMCs are optimized for the local vascular wall ECM stiffness.

## 5. Possible Implications for Disease and Aging

In this review, we discussed mitochondrial structure and function in relation to the cytoskeleton and various mechanical factors influencing cells throughout the body. It is apparent that the dynamics of fission and fusion play a critical role in mitochondrial network organization, which in turn regulates both intra-mitochondrial processes such as ATP production as well as extra-mitochondrial processes such as apoptosis. Mitochondria as a network are also closely associated with various cytoskeletal filamentous networks. Since the cytoskeleton is involved in all mechanosensitive processes, we argued that the mitochondrial network as an organelle is also mechanosensitive and should be viewed as an integral part of the cell’s mechanosensing apparatus. Furthermore, the mitochondrial network is at the center of bi-directional mechanotransduction. On the one hand, the network responds to stretch and its time variations imposed on the mitochondria at the scale of the cell by altering biochemical signaling to produce ATP and ROS at a scale much lower than the network itself. On the other hand, by regulating the cytosolic availability of ATP, ROS, and cytochrome c, the mitochondrial network is able to control whole cell- and possibly tissue- and organ-level processes such as apoptosis, tissue contractility, and organ-level dysfunction.

It has long been appreciated that mitochondria contribute to a variety of diseases as well as aging [19,30,56,96,97,98,99,100,101,102,103,104,105]. Since many diseases involve changes in mechanical factors, it is likely that part of the reason why mitochondrial function becomes abnormal in diseases is the abnormal mechanical environment of the cell. For example, in fibrosis, ECM stiffness increases which in turn influences the cytoskeleton and hence triggers a mitochondrial response as well [101]. Pathologically strong airway smooth muscle contraction is a hallmark feature of asthma. Since muscle contraction requires ATP and because airway wall ECM stiffness also increases in asthma due to remodeling, mitochondria should be involved in the development of asthma [103]. Similarly, aging is accompanied by vascular wall stiffening [106], and both wall stiffness [107] and blood pressure variability [108] increase in hypertension. As we have demonstrated here, both of these factors would have to alter mitochondrial processes and hence this may be a new mechanism through which mitochondria may contribute to aging and hypertension. Expanding on this idea, since nearly all cells in the body experience some mechanical perturbation, we argue that mitochondria should be involved in every disease in which mechanical factors are altered. Currently, the roles of mitochondrial structure and function are underappreciated in mechanobiology, whereas mechanotransduction has not had an impact on medicine. Future studies should thus explore the links between mechanobiology and mitochondria, which we believe may open the door to new understanding and treatment avenues for many human diseases.
